# Multicenter Outcome of Hematopoietic Stem Cell Transplantation for Primary Immune Deficiency Disorders in India

**DOI:** 10.3389/fimmu.2020.606930

**Published:** 2021-01-08

**Authors:** Revathi Raj, Fouzia N. Aboobacker, Satya Prakash Yadav, Ramya Uppuluri, Sunil Bhat, Dharma Choudhry, Vikas Dua, Gaurav Kharya, Neha Rastogi, Mansi Sachdev, Vipin Khandelwal, Venkateswaran Swaminathan, Atish Bakane, Balasubramaniam Ramakrishnan, Biju George

**Affiliations:** ^1^ Department of Pediatric Hematology and Oncology, Apollo Cancer Institutes, Chennai, India; ^2^ Department of Hematology, Christian Medical College, Vellore, India; ^3^ Pediatric Hemato-Oncology & BMT Unit, Medanta The Medicity, Gurgaon, India; ^4^ Department of Pediatric Hematology and Oncology, Narayana Health City, Bangalore, India; ^5^ Department of Pediatric Hematology and Oncology, BLK Super Specialty Hospital, New Delhi, India; ^6^ Department of Pediatric Hematology and Oncology, Fortis Memorial Research Institute, Gurugram, India; ^7^ Department of Pediatric Hematology and Oncology, Indraprastha Apollo Hospital, New Delhi, India; ^8^ Department of Biostatistics, Apollo Hospitals, Chennai, India

**Keywords:** hematopoietic stem cell transplant, primary immune deficiency, conditioning, India, haploidentical

## Abstract

**Background:**

Hematopoietic stem cell transplantation (HSCT) is the curative option for many primary immune deficiency disorders (PID). In the last 5 years, increased awareness, availability of diagnostics based on flow cytometry, genetic testing, improved supportive care, use of reduced toxicity conditioning, and success of haploidentical donor HSCT have improved access to HSCT for children with PID in India. We present results on children with PID who underwent HSCT across India and the factors that influenced outcome.

**Patients and Methods:**

We collected retrospective data on the outcome of HSCT for PID from seven centers. We analyzed the impact of the type of PID, conditioning regimen, time period of HSCT- before or after January 2016, graft versus host disease prophylaxis, cause of mortality and overall survival.

**Results:**

A total of 228 children underwent HSCT for PID at a median age of 12 months (range, 1 to 220 months) with a median follow up of 14.4 months. Infants accounted for 51.3% of the cohort and the male female ratio was 3:1. SCID (25%) and HLH (25%) were the more frequent diagnoses. Matched family donor was available in 36.4% and 44.3% children had a haploidentical HSCT. Reduced and myeloablative conditioning regimens were used with 64% children receiving a treosulfan based conditioning regimen. Peripheral blood stem cells were the predominant graft source at 69.3%. The survival in infants (60.2%) was inferior to children aged over 1 year (75.7% p value = 0.01). Children with Wiskott Aldrich syndrome (74.3%) and chronic granulomatous disease (82.6%) had the best outcomes. The survival was superior in children receiving HSCT from a matched sibling (78%) versus an alternate donor HSCT (61% p value = 0.04). In the cohort transplanted after January 2016 survival improved from 26.8% to 77.5% (p value = 0.00). Infection remains the main cause of mortality at in over 50% children. The 5-year overall survival rate was 68%.

**Conclusion:**

Survival of children with PID undergoing HSCT in India has improved dramatically in last 5 years. Alternate donor HSCT is now feasible and has made a therapeutic option accessible to all children with PID.

## Introduction

Hematopoietic stem cell transplantation (HSCT) is the curative option for many primary immune deficiency disorders (PID). Transplantation for PID requires early recognition and referral by the treating pediatrician. India has a population of over 500 million under the age of 18 years. Data from India is now available on the incidence of PID in India (1, 2). However, a significant number remain undiagnosed and would contribute to the infant and childhood mortality data. The Indian Society for Primary Immune Deficiency (ISPID) was established by a team of physicians involved in children’s care in this field in March 2011 (3). Each year, awareness programs have been arranged systematically across the country through ISPID and International collaborators like Foundation for Primary Immunodeficiency Diseases (FPID) to train pediatricians to recognize PID’s ten warning signs (4). In the last 5 years, centers of excellence for PID have been established to increase access to care across the country with diagnostic and management facilities at a subsidized cost. Access to technical information in this field, knowledge transfer from centers of excellence, and advances in molecular diagnosis has revolutionized care. Mutation screening is now possible for all children with PID. The first HSCT for PID in India was performed in 1998 at Vellore for a child with Wiskott Aldrich Syndrome (WAS). At present, there are over 75 centers in India performing HSCT with increasing numbers of pediatric transplant physicians leading the teams over the last 5 years, with over 1000 allogeneic transplant procedures performed each year. Advances in supportive care and access to generic drugs manufactured within the country have ensured that the programs are sustainable. Several governmental and non-governmental charitable organizations have joined hands to help children with rare diseases. Since January 2016, the number of HSCT for PID in India has increased due to high-resolution HLA typing, flow cytometry, molecular diagnosis, matched unrelated donor registries, haploidentical donors, and reduced toxicity conditioning medications being made available in the country. High-income countries report over 90% survival (5–7) in children undergoing HSCT and the survival % in India and other low to middle income countries ranges from 65% to 70 (8, 9).

We describe here, a multicenter study on the outcome of children with Primary immune deficiency that underwent hematopoietic stem cell transplantation across India.

## Patients and Methods

We retrospectively collected outcome data of children with PID undergoing HSCT at seven HSCT centers from Chennai, Vellore, Delhi, Gurgaon, and Bangalore. Retrospective data included the age at HSCT, sex of the patient, the type of PID, donor and stem cell source, conditioning regimen, and graft-versus-host disease (GVHD) prophylaxis, type of T cell depletion, cause of death and overall survival. The cause of death was analyzed with a focus on infection-related mortality. The overall survival time was defined as the time between transplantation and death from any cause. The overall survival was analyzed with regards to age, sex, diagnosis, and type of PID.

### Statistical Analysis

All normally distributed continuous variables were represented as mean ± SD. Normality of data assessed by Shapiro-Wilk’s test. Comparison of categorical variables was done by either Chi square test or Fisher’s exact test based on the number of observations. An independent sample t- test was used to compare the continuous variables between two groups. Overall survival curve was drawn and estimates were calculated by Kaplan-Meier (KM) method. Log rank test was used to compare survival between factors. Data entry was done in Microsoft Excel 2007. Data analysis and validation was done by IBM SPSS Statistics for Windows Version 25.0, Armonk, NY: IBM corp. All ‘p’ values <0.05 was considered as statistically significant. Minimum outcome data form developed by ISCTR (Indian Stem Cell Registry) was used to collect data. The hospital ethics committee approved of the study and written informed consent was obtained from all patient families.

## Results

### Demographic Data

The patient characteristics with regards to demographic data have been represented in **Table 1**. The median age of the children who underwent HSCT is 12 months with a range from 1 to 220 months. There were more boys than girls in the cohort and girls represented only a quarter of the cohort. Half the children transplanted were under the age of 1 year. The most common indications for HSCT were SCID (n = 55, 24.2%) and familial HLH (n = 61, 26.9%) which accounted for 50% of the children. The other indications were Wiskott Aldrich syndrome (n= 35, 15.4%), chronic granulomatous disease (n = 23, 10.1%), leucocyte adhesion defect (n = 11, 4.5%), X linked agammaglobulinemia (n = 8, 3.5%), Mendelian susceptibility to mycobacterial disease (n = 7, 3.1%), Hyper IgM syndrome (n = 6, 2.6%), Hyper IgE syndrome (n = 5, 2.2%) and common variable immune deficiency (n = 2, 0.9%). Children with rare PIDs such as IPEX syndrome, heme oxygenase deficiency, LRBA deficiency and MHC Class II defects have also been transplanted in India (n = 15, 6.6%). Nearly 80% of the HSCT occurred in the period after January 2016 across all centers in India.

**Table 1:** Patient demographics.

Variable	Frequency
Age at HSCT	
Less than 1 year	117 (51.3%)
Over 1 year	111 (48.7%)
Sex	
Male	165 (72.4%)
Female	63 (27.6%)
Time period	
1998 to 2015	44 (19.3%)
2016 to 2019	184 (80.7%)
Diagnosis	
SCID	55 (24.1%)
HLH	61 (26.8%)
Others	112 (49.1%)
Donor type	
MSD	84 (36.8)
MUD	43 (18.9)
Haplo	101 (44.3)
Conditioning	
Nil	8(3.5%)
RIC	101 (44.3%)
MAC	119 (52.2%)
Chemotherapy	
Nil	8 (3.5%)
Treosulfan	146 (64%)
Other drugs	74 (32.5%)
Stem cell source	
PBSC	158 (69.3%)
Bone marrow	55 (24.1%)
Cord blood	15 (6.6%)
GvHD prophylaxis	
Calcineurin inhibitor	128 (56.1%)
T cell depletion	100 (43.9%)

### Details of HSCT

A fully matched family donor (n = 84, 36.8%) was available only for one third of the children and two thirds had an alternate donor source. Haploidentical family donor was the predominant donor source accounting for 101/228 (44.3%) of the transplantation. Despite access to matched unrelated donor registries, only 43 children (18.9%) had matched unrelated donor transplantation. Reduced intensity conditioning (n = 101, 43.3%) and myeloablative conditioning (n = 119, 52.2%) were equally represented and eight children had an unconditioned HSCT. Treosulfan based conditioning was used in 64% (n = 146) of the transplantation and in over 80% of the infants. Peripheral blood stem cells were used in over two thirds of the patients with a small cohort in the cord transplantation group. Notably, no cord blood transplantation has been performed after January 2016 in the country.

Graft versus host disease prophylaxis consisted predominantly of calcineurin inhibitors in 128 children (56.1%). Haploidentical HSCT was performed in 100 children (42.9%) with the use of post transplant cyclophosphamide in 57/100 (57%) and TCR alpha beta depletion in 43/100 (43%) children.

### Variables Affecting HSCT Outcome

**Figure 1:**
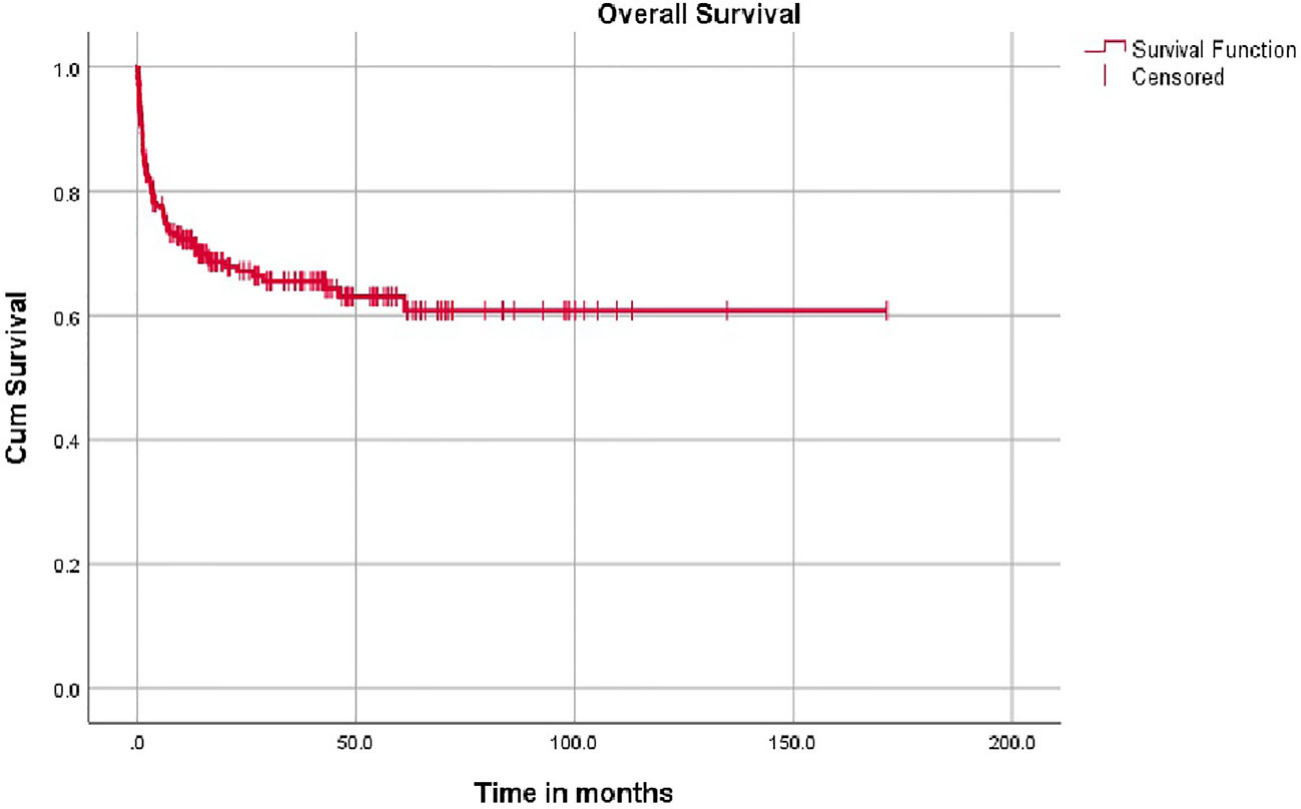
Overall survival.

The impact of the demographics, donor source, conditioning regimen, and GvHD prophylaxis and have been represented in **Table 2**. The survival was superior in age over 1 year and the outcomes were similar in male and female children. Children with SCID had an inferior survival (50.9%) compared to children with Wiskott Aldrich syndrome, chronic granulomatous disease (CGD) and Hyper IgM (74.3%, 82.6% and 100% respectively). The most significant finding was the increase in the number of PID transplants from January 2016 in the country and the improved survival for children transplanted after January 2016 as shown in **Figure 1**.

**Table 2:** Transplant characteristics and impact on survival.

Variable	Mean survival time in months (95% CI)	Overall survival	P value (log rank test)
Age at HSCT			
Less than 1 year	95.2 (78.1–112.3)	60.2%	0.022
Over 1 year	77.8 (68.7–86.9)	75.7%	
Sex			
Male	102.4 (87.5–117.3)	64.6%	0.157
Female	86.9 (74.9–99)	77.0%	
Time period			
1998 to 2015	18.8 (3.6–33.9)	26.8%	0.0001
2016 to 2019	101.3 (4.6–92.1)	77.5%	
Diagnosis			
SCID	55.3 (40.2–70.4)	50.9%	0.009
HLH	65.8 (53.4–78.1)	68.9%	
Others	123.8 (108.1–139.5)	73%	
Donor type			
MSD	128.5 (111.4–145.8)	78%	0.023
MUD	55.7 (39–72.3)	60.5%	
Haplo	43.2 (36.3–50.1)	62.8%	
Conditioning			
Nil	41.9 (19.5–64.3)	62.5%	0.497
RIC	62.7 (52.6–72.9)	64%	
MAC	115.1 (98.4–131.8)	71.4%	
Chemotherapy			
Nil	37.4 (15.8–58.9)	55.6%	0.848
Treosulfan	69.6 (60.7–78.5)	69.2%	
Other drugs	106.4 (85.6–127.2)	65.7%	
Stem cell source			
PBSC	90 (78.8–101.3)	70.2%	0.038
Bone marrow	109.2 (82.2–136.3)	69.8%	
Cord blood	44.5 (17.6–71.3)	40.0%	
GvHD prophylaxis			
Calcineurin inhibitor	114.3 (98.8–129.7	70%	0.232
T cell depletion	44.2 (37.3–51.1)	64%	

**Figure 2:**
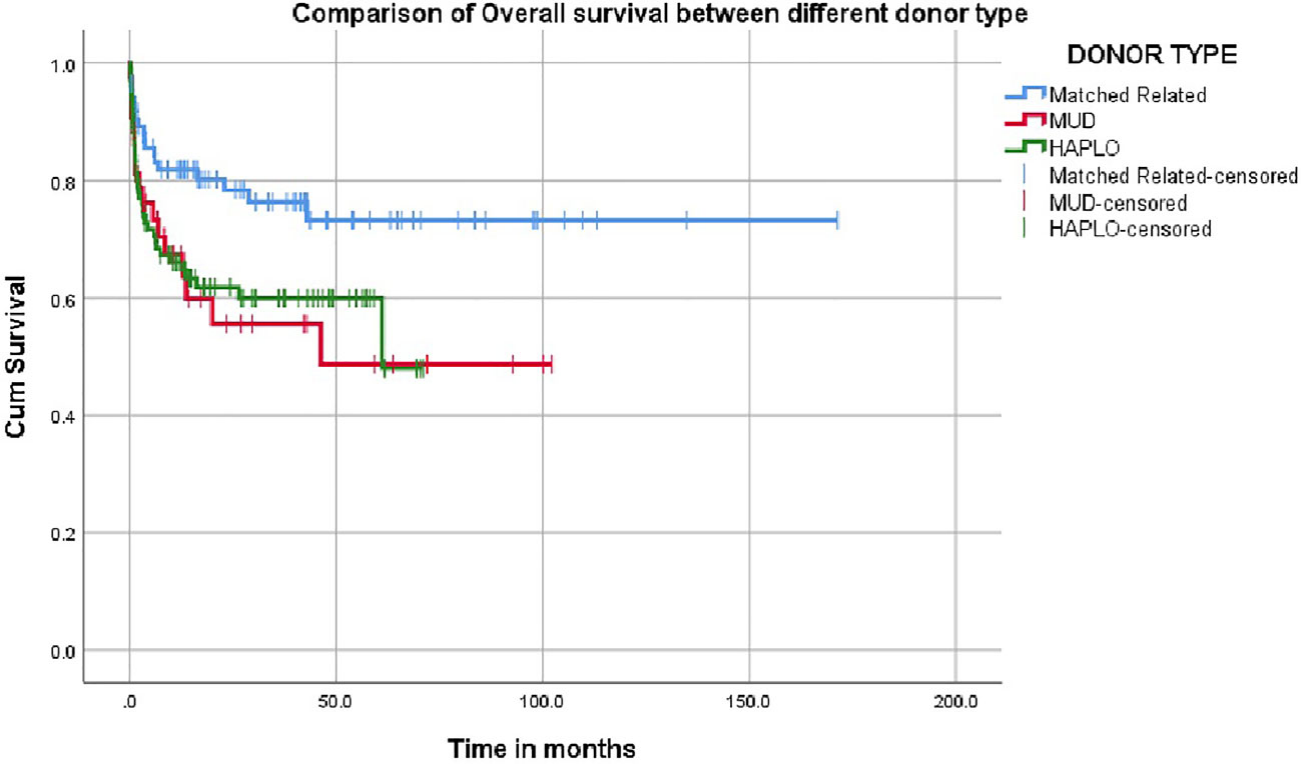
Impact on donor source on survival.

**Figure 3:**
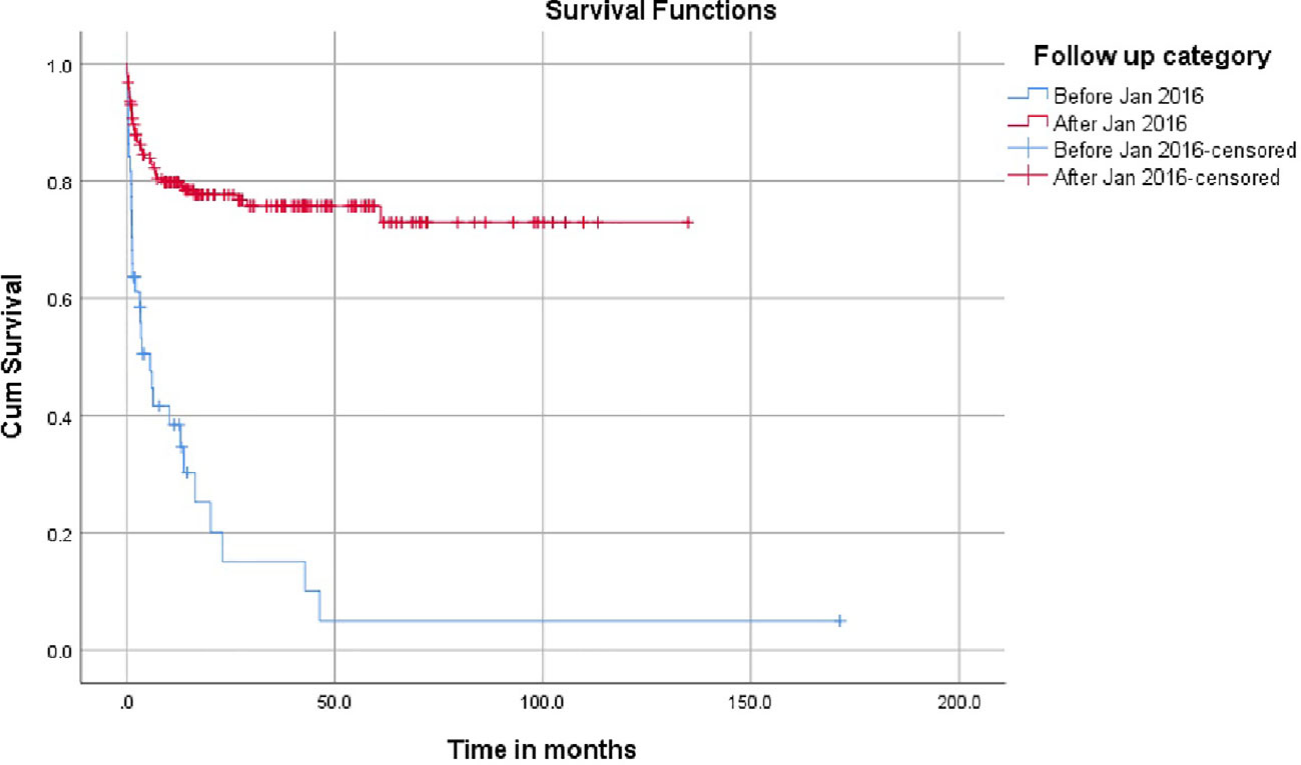
Impact on time period on survival.

The overall survival was superior for children undergoing HSCT from matched family donors at 78% compared to alternate donor HSCT at 61% (**Figure 2**). In this mixed cohort, there was no difference in survival between myeloablative and reduced-intensity conditioning regimens. Cord as a source of stem cells resulted in poor survival of 40%. The survival in TCR alpha beta depletion 63.9% was equal to post-transplant cyclophosphamide at 64.9% (p value = 0.8) (**Figure 3**).

Infection was the leading cause of mortality at 53.9%, followed by graft versus host disease (GVHD) at 17%, graft rejection at 10.6%, and regimen related toxicity at 13.1%. A total of 11 children underwent a second HSCT for graft rejection and 8 children are alive following a second HSCT.

The most frequent presentation of disseminated BCG was a painful swelling or induration at the BCG vaccination site with axillary lymphadenitis. The other manifestations included intermittent fever, subcutaneous nodules, lesions in the liver and spleen, miliary involvement of the lung and osteomyelitis. These children were treated with a three drug regimen including isoniazid, ethambutol and levofloxacin during the transplant period. Calcineurin inhibitors interacted with rifampicin and this drug was introduced only when the immunosuppression was stopped. BCG infection is inherently resistant to pyrazinamide.

The overall mean survival time is 108 months (96.2–120.8). The 5 year overall survival rate is 68% (62%–74%) with a median follow up is 14.4 months (3.3–42.6) (IQR). Around 61% of patients had follow up of 2 years and 87.6% of patients had follow up of 5 years. Event free survival data was calculated on 118 patients with comprehensive data set was available and was 53.3%.

## Discussion

Advances in the field of HSCT have resulted in improved survival for children with PID in high-income countries (10, 11). The impact of working together as a team has been demonstrated by the Primary Immune Deficiency Treatment Consortium (PIDTC) in North America, which has supported the diagnosis and management and research in the field of PID (12–15). Similar results have been seen in the EBMT (16–18) and Latin American groups (19) with data from focused groups showing improvement in overall survival. Significant challenges in early diagnosis, supportive care and financial constraints need to be overcome in low and middle income countries as reported by groups from Brazil, Jordan, China, Iran and Turkey (20–26).

In India, the field of PID has come up in leaps and bound over the past 5 years. The formation of the Indian Society for Primary Immune Deficiency (ISPID) resulted in regular meetings conducted with the help of experts from around the world. Training of physicians involved in the diagnosis and management of PID and knowledge transfer have resulted in improved survival seen from January 2016 (27). The progress has been slow but steady, and the introduction of haploidentical HSCT allowed many children to be transplanted (28–31). HSCT in India is poised at a critical juncture where collaborative trails and research is feasible with the formal launch of ISBMT – Indian Society for Blood and Marrow Transplantation. New centers focused on HSCT for PID have been established and have reported outcomes on 21 procedures over the last 2 years with an overall survival of 60% in critically ill children requiring haploidentical HSCT (oral communication Battad et al.).

This is the first report of a multicenter data on HSCT for PID in India. All children with PID now have access to molecular diagnosis and treatment (32, 33). Although only a third of the children have a matched family donor and unrelated registries like DATRI have a donor database of over 600,000 volunteers in the country, and this has made MUD transplants a reality (34). Unrelated cord transplantation has now been phased out in all HSCT centers as delayed engraftment results in unacceptably high transplant related mortality due to bacterial infections (oral communication ISBMT). Haploidentical HSCT with PTCY and TCR alpha beta depletion show promising results although the cost of HSCT is high with TCR alpha beta depletion (35, 36). Haploidentical HSCT with TCR alpha beta depletion will continue to hold a place in HSCT performed for infants and children with significant comorbidity despite the high costs involved. These numbers would also increase once newborn screening programs for SCID are established in the country.

Primary immunodeficiencies (PID) are disorders resulting from mutations in genes involved in immune host defense and immunoregulation. Replacement of hematopoietic stem cells with full or partial ablation of the recipient’s marrow with chemotherapy allows stable engraftment of donor-derived stem cell. The conditioning regimen is the key to reduce the risks of graft rejection and graft versus host disease (37–39). The use of treosulfan in 64% of the myeloablative conditioning has reduced mortality from sinusoidal obstruction syndrome. Nearly 70% of the infants undergoing HSCT were treated on the treosulfan. There are no laboratories to perform busulfan pharmacokinetics, and hence targeted busulfan therapy is not feasible and busulfan pharmacokinetics is unpredictable in infants. Generic forms of busulfan, melphalan, and thiotepa are now available in the country, which helps reduce the cost of conditioning drugs.

There has been a marked increase in the incidence of multidrug resistant bacterial infections since 2012 in the country. Multidrug resistant bacterial infections, late diagnosis and delayed referral contribute significantly to inferior outcomes in our country. Most infants have required intensive care support before transplantation due to infection as reported in published literature (40). Newer antifungal agents, such as anidulafungin and posaconazole, have helped combat fungal infections in this cohort. Ganciclovir, valganciclovir, and cidofovir are available for the treatment of CMV infection. Disseminated BCG remains a challenge as all babies are vaccinated with BCG at birth. Over 80% of the children with PID required prolonged antitubercular drugs to eliminate BCG infection with steroid cover during immune reconstitution syndrome (IRIS).

Graft versus host disease resulted in mortality in 17% of the children and graft rejection in 10.6%. Newer therapies like ruxolitinib and extracorporeal photopheresis are now available and will help reduce mortality from graft versus host disease. Follow-up of these children requires optimal communication with the shared care pediatrician for vaccination, early treatment of infections, and evaluation for late effects. Graft rejection, the need for immunoglobulin replacement and chronic graft versus host disease and poor immune reconstitution resulted in poor quality of life in about 15% of our survivors.

Female children comprised only 27.6% of the cohort and this reflects gender inequality issues that have persisted for decades in the community where expensive therapy like HSCT are preferentially offered to a male child. The survival in infants with SCID has been poor due to late presentation due to delayed diagnosis and lack of financial support. HSCT for children with XLA and CVID is on the increase in the country as lifelong immunoglobulin replacement is a major therapeutic challenge in all low income countries (41, 42). Newer options like gene therapy require specialized infrastructure and funding. Several groups in India are now engaged in gene therapy research to provide a financially viable option for families with rare genetic disorders (43, 44).

## Conclusion

The survival of children with PID undergoing HSCT in India has improved dramatically in last 5 years. Alternate donor HSCT has made HSCT accessible to children with PID. The overall survival of 68% is lower than centers in high-income countries. There is an urgent need to improve our services in early diagnosis, newborn screening, supportive care and the introduction of novel technologies like indigenous gene therapy and gene editing.

## Data Availability Statement

The original contributions presented in the study are included in the article/supplementary materials. Further inquiries can be directed to the corresponding author/s.

## Ethics Statement

The studies involving human participants were reviewed and approved by the Apollo Speciality Hospital, Chennai, India. Written informed consent to participate in this study was provided by the participants’ legal guardian/next of kin.

## Author Contributions

All authors listed have made a substantial, direct, and intellectual contribution to the work and approved it for publication. BR helped with data analysis and all other authors delivered patient care and contributed data. 

## Conflict of Interest

The authors declare that the research was conducted in the absence of any commercial or financial relationships that could be construed as a potential conflict of interest.
